# Antiplatelet effect of ticagrelor with aspirin in acute minor stroke and transient ischemic attack stratified by *CYP2C19* metabolizer status: subgroup analysis of the PRINCE trial

**DOI:** 10.18632/aging.202366

**Published:** 2020-12-19

**Authors:** Mengyuan Zhou, Weiqi Chen, Yuesong Pan, Yi Lin, Xia Meng, Xingquan Zhao, Liping Liu, Jinxi Lin, Hao Li, Yongjun Wang, Yilong Wang

**Affiliations:** 1 Department of Neurology, Beijing Tiantan Hospital, Capital Medical University, Beijing, China; 2 China National Clinical Research Center for Neurological Diseases, Beijing, China; 3 Advanced Innovation Center for Human Brain Protection, Capital Medical University, Beijing, China; 4 Beijing Key Laboratory of Translational Medicine for Cerebrovascular Disease, Beijing, China; 5 Department of Neurology and Institute of Neurology, First Affiliated Hospital, Fujian Medical University, Fuzhou, China

**Keywords:** antiplatelet, clopidogrel, high platelet reactivity, inhibition of platelet aggregation, ticagrelor

## Abstract

Studies on antiplatelet effect of ticagrelor/aspirin and clopidogrel/aspirin in patients with acute minor stroke and transient ischemic attack (TIA) stratified by *CYP2C19* metabolizer status is limited. We gained data from the Platelet Reactivity In Non-disabling Cerebrovascular Events study. Platelet reactivity was tested at baseline, 2 hours, 24 hours, 7 days and 90 days after initial dose, including high on-treatment platelet reactivity (HOPR), which was defined as P2Y12 reaction unit >208, and percentage inhibition of platelet aggregation (IPA). A total of 365 patients were included. There were 199 (54.5%) individuals classified as carriers of *CYP2C19* loss-of-function alleles. For carriers and non-carriers, the proportions of HOPR were significantly lower in those with ticagrelor/aspirin compared with those with clopidogrel/aspirin at 2 hours, 24 hours, 7 days, respectively (all p<0.05). IPA was higher at all time points except at baseline in patients with ticagrelor/aspirin compared with those with clopidogrel/aspirin in both carriers and non-carriers of *CYP2C19* lose-of-function alleles (all p<0.05). Our findings showed that ticagrelor/aspirin therapy possessed greater platelet inhibition and more rapid onset in platelet inhibition compared with clopidogrel/aspirin therapy both in carriers and non-carriers of *CYP2C19* lose-of-function alleles with acute minor stroke or TIA.

## INTRODUCTION

The risk of stroke recurrence is high after acute minor stroke or transient ischemic attack (TIA). [1] The Clopidogrel with Aspirin in Acute Minor Stroke or Transient Ischemic Attack study showed that combination of clopidogrel and aspirin with 24 hours could reduce the risk of stroke in 90 days without increasing the risk of hemorrhage. [2] However, the subsequent analysis found that the application of clopidogrel and aspirin compared with aspirin alone reduced the risk of recurrent stroke only in patients who were non-carriers of the *CYP2C19* loss-of-function (LOF) alleles. [3] The polymorphisms of the *CYP2C19* gene have been identified to play a significant role in the metabolism of clopidogrel. [4] Previous study showed that about 40-60% Asian participants were carriers of *CYP2C19* LOF alleles, who could not benefit from clopidogrel. [3]

Ticagrelor is a new, oral, direct-acting, and reversible P2Y12 ADP receptor blocker which does not require metabolic activation. Several studies showed that ticagrelor possessed a faster and greater antiplatelet effect and was more effective than clopidogrel irrespective of variants in the *CYP2C19* genotype in cardiovascular disease. [5, 6] The Acute Stroke or Transient Ischemic Attack Treated with Aspirin or Ticagrelor and Patient Outcomes (SOCRATES) trial was the first study to compare the efficacy and safety of ticagrelor versus aspirin in patients with acute ischemic stroke or TIA, but did not find a significant result. [7] However, the subgroup analysis of the SOCRATES trial [8] indicated that ticagrelor might be superior to aspirin at preventing atherosclerotic origin cerebrovascular events. Whether patients can benefit from more intensive antiplatelet therapy-combination of ticagrelor and aspirin remains controversial. Recently, the Platelet Reactivity In Non-disabling Cerebrovascular Events (PRINCE) study reported that patients with minor stroke or TIA treated with ticagrelor/aspirin had a lower proportion of high on-treatment platelet reactivity (HOPR), especially in carriers of the *CYP2C19* LOF alleles compared with those treated with clopidogrel/aspirin. [9]

This sub-analysis aimed to investigate the effect of ticagrelor/aspirin on HOPR and inhibition of platelet aggregation (IPA) during study time course stratified by *CYP2C19* metabolizer status compared with clopidogrel/aspirin.

## RESULTS

**Figure 1:**
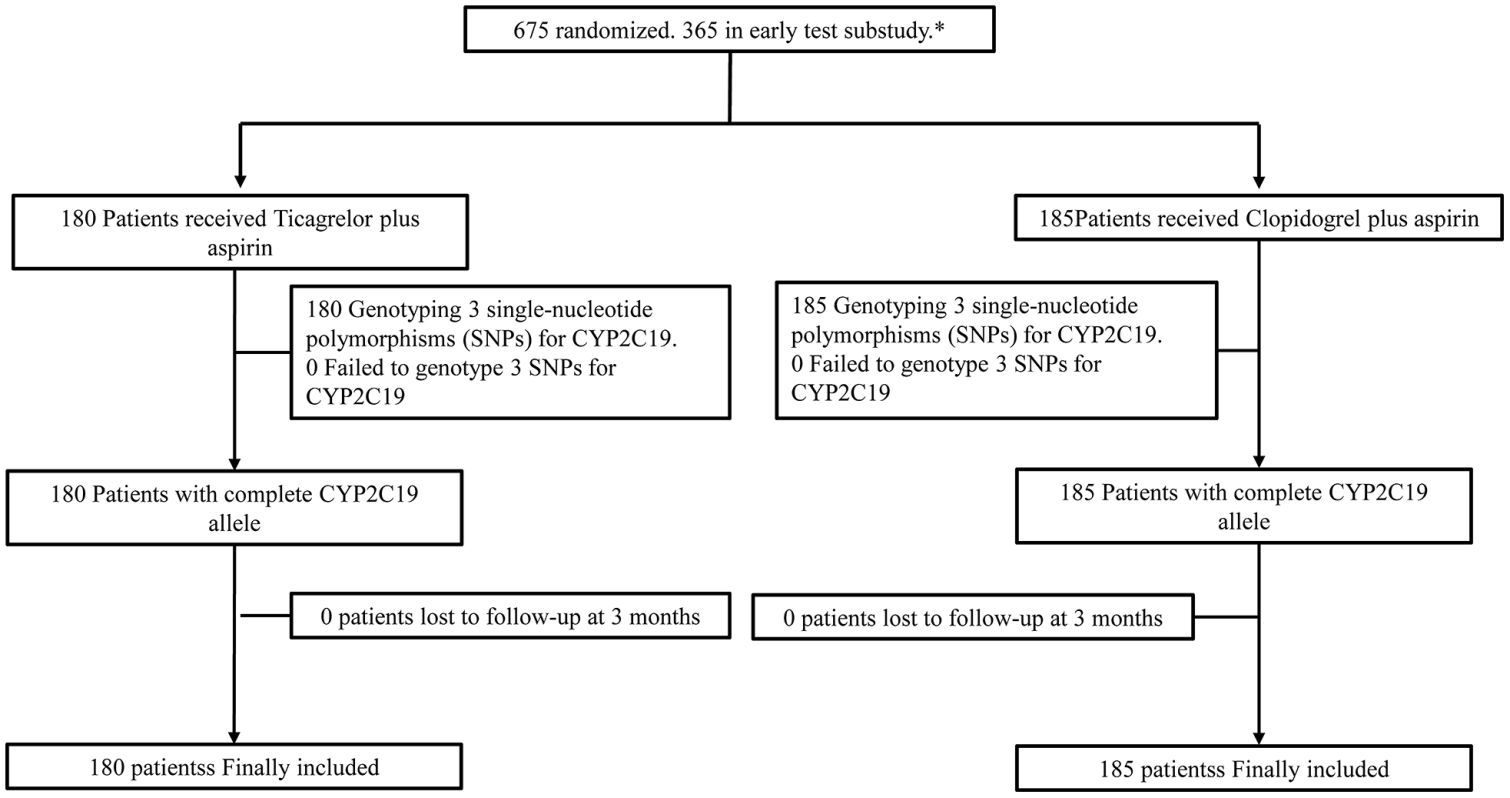
**Flow chart.**

Study flow was shown in Figure 1. We conducted valid measurements in 376 and 373 patients for VerifyNow P2Y12 assay at 2 hours and 24 hours after first dose respectively. A total of 365 out of 675 patients with acute minor stroke and TIA were finally included in our early test sub-study with no patient missed *CYP2C19* genotype data or lost to follow up. Patients included tended to be older, have a history of dyslipidemia, use statin and aspirin before randomization (Table 1). Among the 365 included patients, 105 (28.8%) of them were female, the average age was 61.7±8.5 years, 199 (54.5%) of them were classified as carriers of *CYP2C19* LOF alleles, 180 (49.3%) of them received ticagrelor/aspirin therapy and 185 (50.7%) of them received clopidogrel/aspirin therapy (Table 2). Baseline characteristics between LOF allele carriers and non-carriers were well balanced.

**Table 1:** Baseline characteristics of patients included and excluded.

**Characteristic**	**Included (N=365)**	**Excluded (N=310)**	**p**
Age (years), mean (SD)	61.7±8.5	59.7±8.8	**0.002**
Female sex, n (%)	105 (28.8%)	76 (24.5%)	0.21
Systolic blood pressure (mmHg), mean (SD)	153.8±21.6	153.5±22.2	0.91
Diastolic blood pressure (mmHg), mean (SD)	88.0±13.0	89.2±12.9	0.24
Body-mass index (kg/m^2^) ^a^, mean (SD)	24.9±3.8	25.1±3.8	0.45
Pulse rate (beats/min), mean (SD)	76.2±10.7	75.0±11.0	0.14
Medical history (%)			
Hypertension	229 (62.7%)	182 (58.7%)	0.29
Dyslipidemia	29 (8.0%)	12 (3.9%)	**0.03**
Diabetes mellitus	87 (23.8%)	77 (24.8%)	0.76
Ischemic stroke	68 (18.6%)	53 (17.1%)	0.60
TIA	11 (3.0%)	7 (2.3%)	0.54
Coronary artery disease	18 (4.9%)	33 (10.7%)	**0.005**
Known atrial fibrillation	1 (0.3%)	3 (1.0%)	0.51
Flutter valvular heart disease	0 (0.0%)	1 (0.3%)	0.28
Pulmonary embolism	0 (0.0%)	0 (0.0%)	-
Smoking status (%)			
Non-smoker	179 (49.0%)	126 (40.7%)	0.07
Current smoker	158 (43.3%)	161 (51.9%)	
Ex-smoker	28 (7.7%)	23 (7.4%)	
Drug use before randomization (%)			
Proton-pump inhibitor	3 (0.8%)	2 (0.7)	0.79
Statin	44 (12.1%)	22 (7.1%)	**0.03**
Aspirin	90 (24.7%)	56 (18.1%)	**0.04**
Clopidogrel	10 (2.7%)	5 (1.6%)	0.32
Ticagrelor	0 (0.0%)	0 (0.0%)	-
Mean time to randomization after onset of symptoms (h), mean (SD)	14.0±6.6	14.3±6.8	0.49
Time to randomization after onset of symptoms (%)			0.43
<12 hr	148 (40.6%)	135 (43.6%)	
≥12 hr	217 (59.5%)	175 (56.5%)	
Qualifying event (%)			0.67
Minor stroke	307 (84.1%)	257 (82.9%)	
TIA^b^	58 (15.9%)	53 (17.1%)	
Baseline ABCD^2^ score among patients with TIA as qualifying event^c^			0.33
Median	5.0	5.0	
Interquartile range	4.0-5.0	4.0-5.0	
SSS-TOAST stroke subtype (%)^d^			0.17
Large-artery atherosclerosis	156 (50.8%)	148 (57.6%)	
Cardioaortic embolism	7 (2.3%)	6 (2.3%)	
Small-artery occlusion	120 (39.1%)	93 (36.2)	
Other causes	13 (4.2%)	3 (1.2%)	
Undetermined causes	11 (3.6%)	7 (2.7%)	
Unkown	7 (2.3%)	2 (0.8%)	
Unclassified	4 (1.3%)	5 (1.9%)	

^a^The body-mass index is the weight in kilograms divided by the square of the height in meters.

^b^TIA indicates transient ischemic attack.

^c^ABCD^2^ stroke risk scores range from 0 to 7, with higher scores meaning higher risk; data provided in the table only for the group of 111 patients whose qualifying event was TIA for inclusion in the trial.

^d^SSS-TOAST stroke subtype=Stop Stroke Study Trial of Org 10172 in Acute Stroke Treatment stroke aetiology classification (supplementary appendix, SSS-TOAST classification criteria); data provided in the table are only for the group of 564 patients whose qualifying event was minor stroke for inclusion in the trial

**Table 2:** Baseline characteristics of patients received ticagrelor/aspirin and clopidogrel/aspirin stratified by CYP2C19 metabolizer status.

**Characteristic**	**Carrier**				**Non-carrier**			
	**Total (n = 199)**	**Ticagrelor/aspirin (n = 100)**	**Clopidogrel/aspirin (n =99)**	**p**	**Total (n = 166)**	**Ticagrelor/aspirin (n = 80)**	**Clopidogrel/aspirin (n = 86)**	**p**
Age (years), mean (SD)	61.3±9.1	61.8±8.6	60.8±9.5	0.44	62.3±7.8	62.5±7.5	62.1±8.1	0.72
Female sex, n (%)	48 (24.1%)	19 (19%)	29(29.3%)	0.09	57 (34.3%)	33 (41.3%)	24 (27.9%)	0.07
Systolic blood pressure (mmHg), mean (SD)	153.2±21.9	153.5±21.6	153.0±22.3	0.86	154.3±21.4	152.6±24.2	155.9±18.4	0.33
Diastolic blood pressure (mmHg), mean (SD)	88.3±13.2	88.6±12.4	88.1±14.1	0.79	87.7±12.7	86.4±12.8	88.8±12.5	0.22
Body-mass index (kg/m^2^)^a^, mean (SD)	25.1±3.7	24.9±2.7	25.3±4.5	0.40	24.7±3.8	24.9±4.0	24.5±3.7	0.45
Pulse rate (beats/min), mean (SD)	77.0±10.0	75.5±8.5	77.9±11.3	0.10	75.7±11.5	76.6±10.3	74.9±12.5	0.35
Medical history (%)								
Hypertension	124	67(67%)	57(57.6%)	0.17	105	48 (60%)	57 (66.3%)	0.40
Dyslipidemia	16	10(10%)	6(6.1%)	0.31	13	6 (7.5%)	7 (8.1%)	0.02
Diabetes mellitus	47	24(24%)	23(23.2%)	0.90	40	18 (22.5%)	22 (25.6%)	0.64
Ischemic stroke	36	18(18%)	18(18.2%)	0.97	32	12 (15%)	20 (23.3%)	0.18
TIA	6	1(1%)	5(5.1%)	0.09	5	3 (3.8%)	2 (2.3%)	0.59
Coronary artery disease	9	4(4%)	5(5.1%)	0.72	9	6 (7.5%)	3 (3.5%)	0.25
Known atrial fib rillation	1	0(0%)	1(1.0%)	-	0	0	0	-
Flutter valvular heart disease	0	0	0	-	0	0	0	-
Pulmonary embolism	0	0	0	-	0	0	0	-
Smoking status (%)				0.72				0.80
Non-smoker	92	44(44%)	48(48.5%)		87	44 (55%)	43 (50%)	
Current smoker	86	44(44%)	42(42.4%)		72	33 (41.3%)	39 (45.3%)	
Ex-smoker	21	12(12%)	9(9.1%)		7	3 (3.8%)	4 (4.7%)	
Drug use before randomization (%)								
Proton-pump inhibitor	3	1(1%)	2(2.0%)	0.55	0	0	0	-
Statin	25	16(16%)	9(9.1%)	0.14	19	8 (10%)	11 (12.8%)	0.57
Aspirin	50	29(29%)	21(21.2%)	0.21	40	22 (27.5%)	18 (20.9%)	0.32
Clopidogrel	6	1(1%)	5(5.1%)	0.10	4	2 (2.5%)	2 (2.3%)	0.94
Ticagrelor	0	0	0	-	0	0	0	-
Mean time to randomization after onset of symptoms (h), mean (SD)	13.9±6.6	14.5±6.6	13.3±6.5	0.22	14.1±6.6	13.7±6.6	14.4±6.6	0.47
Time to randomization after onset of symptoms (%)				0.07				0.30
<12 hr	78	33(33%)	45(45.5%)		96	43 (53.8%)	53 (61.6%)	
≥12 hr	121	67(67%)	54(54.5%)		70	37 (46.3%)	33 (38.4%)	
Qualifying event (%)	199			0.10	166			0.87
Minor stroke	173	83(83%)	90(90.9%)		134	65 (81.3%)	69 (80.2%)	
TIA^b^	26	17(17%)	9(9.1%)		32	15 (18.8%)	17 (19.8%)	
Baseline ABCD^2^ score among patients with TIA as qualifying event^c^				0.30				0.23
Median	5.0	5.0	5.0		5.0	5.0	4.0	
Interquartile range	4.0-6.0	4.0-5.0	4.0-7.0		4.0-5.0	4.0-6.0	4.0-5.0	
SSS-TOAST stroke subtype (%)^d^				0.37				0.47
Large-artery atherosclerosis	90	42 (24.3%)	48 (27.8%)		66	36 (26.9%)	30 (22.4%)	
Cardioaortic embolism	4	3 (1.7%)	1 (0.6%)		3	2 (1.5%)	1 (0.7%)	
Small-artery occlusion	62	33 (19.1%)	29 (16.8%)		58	23 (17.2%)	35 (26.1%)	
Other causes	10	3 (1.7%)	7 (4.0%)		3	2 (1.5%)	1 (0.7%)	
Undetermined causes	7	2 (1.2%)	5 (2.9%)		4	2 (1.5%)	2 (1.5%)	
Unknown	4	0 (0.0%)	4 (2.3%)		3	1 (0.7%)	2 (1.5%)	
Unclassified	3	2 (1.2%)	1 (06%)		1	1 (0.7%)	0 (0.0%)	

^a^The body-mass index is the weight in kilograms divided by the square of the height in meters.

^b^TIA indicates transient ischemic attack.

^c^ABCD^2^ stroke risk scores range from 0 to 7, with higher scores meaning higher risk; data provided in the table only for the group of 111 patients whose qualifying event was TIA for inclusion in the trial.

^d^SSS-TOAST stroke subtype=Stop Stroke Study Trial of Org 10172 in Acute Stroke Treatment stroke aetiology classification (supplementary appendix, SSS-TOAST classification criteria); data provided in the table are only for the group of 564 patients whose qualifying event was minor stroke for inclusion in the trial.

### High on-treatment platelet reactivity

**Figure 2:**
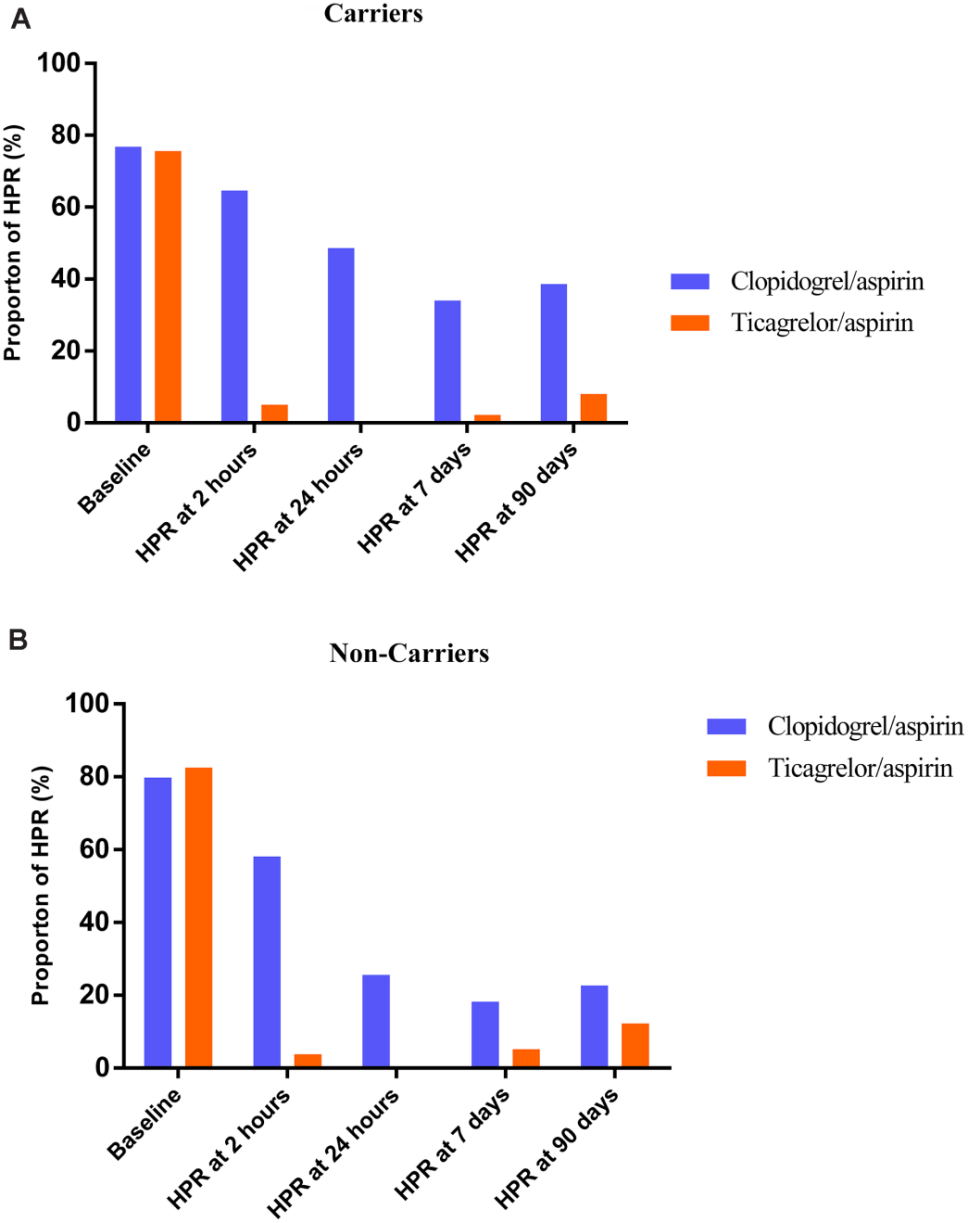
**Effect of Ticagrelor/aspirin therapy compared with Clopidogrel/aspirin therapy on high on-treatment platelet reactivity (HOPR) during study time course stratified by metabolizer status.** (**A**) Carriers; (**B**) Non-carriers.

For carriers and non-carriers, proportions of HOPR were significantly lower during ticagrelor/aspirin therapy compared with clopidogrel/aspirin therapy at 2 hours, 24 hours, 7 days, respectively (all p<0.05), which were similar at baseline (Table 3). The proportion of HOPR in the ticagrelor/aspirin group was only significantly lower than that in the clopidogrel/aspirin group in carriers, while no significant difference was found in proportions of HOPR between ticagrelor/aspirin group and clopidogrel/aspirin group in non-carriers at 90 days (HOPR proportion among carriers, 8.0% with ticagrelor/aspirin therapy vs 38.6% with clopidogrel/aspirin therapy; risk ratio (RR), 0.21; 95% confidence intervals (CI), 0.09-0.41; p<0.001; HOPR proportion among non-carriers, 12.3% with ticagrelor/aspirin therapy vs 22.7% with clopidogrel/aspirin therapy; RR, 0.54; 95% CI, 0.25-1.11; p=0.11; p=0.37 for interaction). The Ticagrelor/aspirin therapy was more effective in reducing proportion of HOPR independent of *CYP2C19* metabolizer status and seemed to eliminate HOPR at 24 hours in both carriers and non-carriers (Figure 2).

**Table 3:** Effect of ticagrelor/aspirin as compared with clopidogrel/aspirin on high on-treatment platelet reactivity (HOPR) during study time course stratified by metabolizer status.

	**Carriers^a^**				**Non-carriers^b^**				
	**Ticagrelor/aspirin**	**Clopidogrel/aspirin**	**Risk ratio (95% CI)**	**p**	**Ticagrelor/aspirin**	**Clopidogrel/aspirin**	**Risk ratio (95% CI)**	**p**	**p value for interaction**
Baseline	75/99 (75.6%)	76/99(76.8%)	0.99(0.84-1.16)	0.87	66/80(82.5%)	67/84(79.8%)	1.03(0.89-1.21)	0.65	0.86
HOPR^c^ at 2 hours	5/100(5.0%)	64/99(64.6%)	0.08(0.03-0.16)	<0.001	3/80(3.8%)	50/86(58.1%)	0.07(0.02-0.17)	<0.001	0.86
HOPR at 24 hours	0/100(0%)	48/99(48.6%)	-	1.0	0/80(0%)	22/86(25.6%)	-	1.0	0.26
HOPR at 7 days	2/93(2.2%)	33/97(34.0%)	0.06(0.01-0.20)	<0.001	4/77(5.2%)	15/82(18.3%)	0.28(0.08-0.74)	0.02	0.37
HOPR at 90 days	7/88(8.0%)	32/83(38.6%)	0.21(0.09-0.41)	<0.001	9/73(12.3%)	17/75(22.7%)	0.54(0.25-1.11)	0.11	0.37

^a^Loss-of-function allele carriers, defined as patients with at least one CYP2C19 loss-of-function allele (ie, *2 or *3): *1/*2, *1/*3, *2/*2, *2/*3, *3/*3, *2/*17, or *3/*17.

^b^Loss-of-function non-carriers were defined as patients with no CYP2C19 loss-of-function allele: *1/*1, *1/*17, or *17/*17.

^c^HOPR, high on-treatment platelet reactivity, defined as the P2Y12 reaction unit (PRU) >208 measured using the VerifyNow P2Y12 assay.

### Inhibition of platelet aggregation

**Figure 3:**
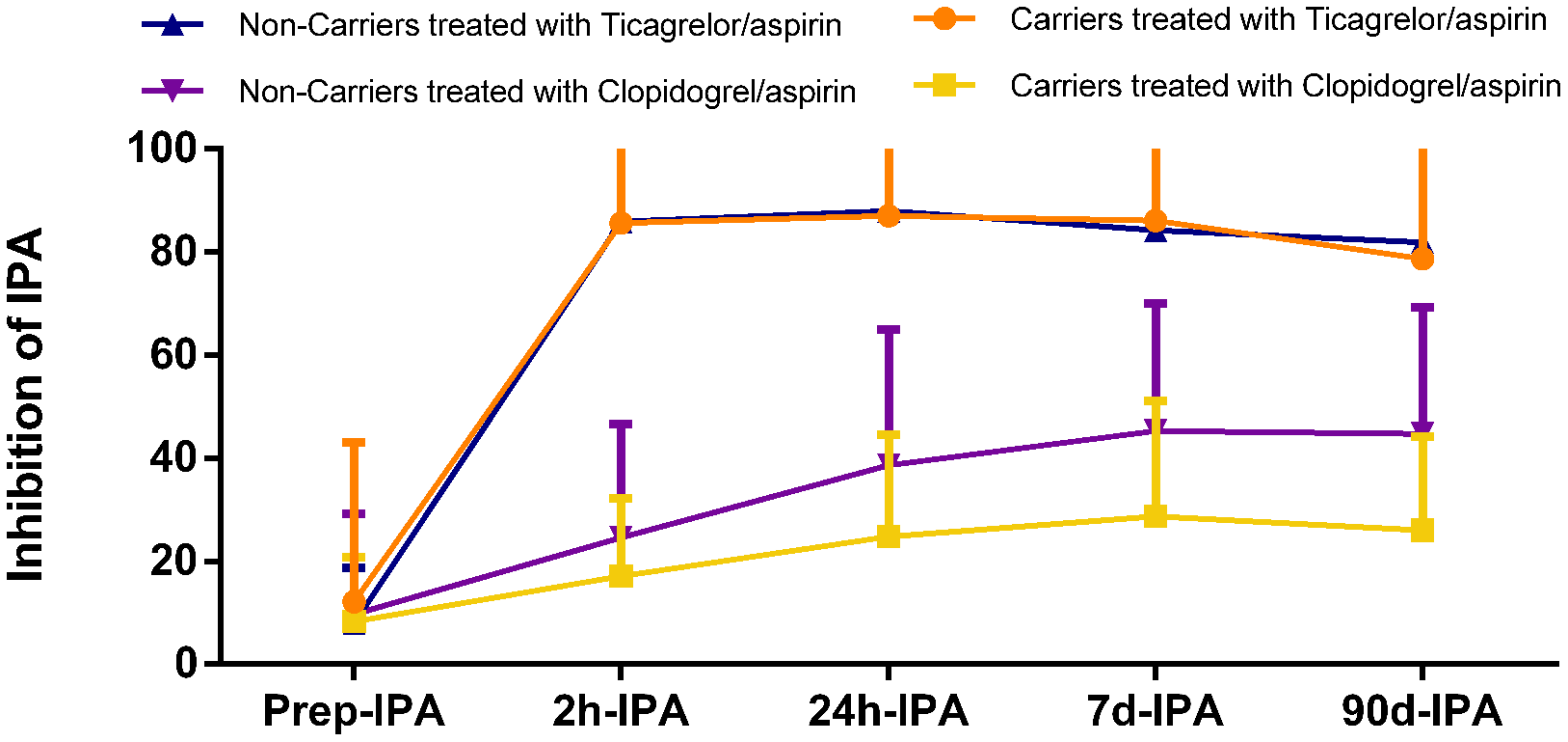
**Inhibition of platelet aggregation (IPA) of Ticagrelor/aspirin therapy as compared with Clopidogrel/aspirin therapy during study time course stratified by metabolizer status.**

IPA was higher at all time points except at baseline in patients treated with ticagrelor/aspirin therapy compared with those treated with clopidogrel/aspirin therapy in both carriers and non-carriers of *CYP2C19* LOF alleles (all p<0.001 for 2h, 24h, 7d and 90d-IPA, respectively; Table 4). IPA was similar in carriers and non-carriers treated with ticagrelor/aspirin therapy, while was different between carriers and non-carriers treated with clopidogrel/aspirin therapy (Figure 3).

**Table 4:** Inhibition of platelet aggregation (IPA) of ticagrelor/aspirin as compared with clopidogrel/aspirin treatment during study time course stratified by metabolizer status.

	**Carriers^a^**				**Non-carriers^b^**				**p value for interaction**
	**Ticagrelor/aspirin**	**Clopidogrel/aspirin**	**β (95% CI)**	**p**	**Ticagrelor/aspirin**	**Clopidogrel/aspirin**	**β (95% CI)**	**p**	
Prer-IPA	12.1 ± 30.9	8.3 ± 12.5	3.77 (-6.20-13.73)	0.46	7.9 ± 10.9	9.7 ± 19.6	-1.78 (-9.24-5.68)	0.64	0.42
2h-IPA	85.6 ± 17.5	17.1 ± 15.2	68.54 (63.27-73.79)	**<0.001**	85.9 ± 19.2	24.6 ± 22.1	61.35 (54.51-68.19)	**<0.001**	0.10
24h-IPA	87.0 ± 15.9	24.8 ± 19.8	62.13 (56.90-67.35)	**<0.001**	88.0 ± 13.0	38.6 ± 26.4	49.40 (42.95-55.85)	**<0.001**	0.003
7d-IPA	86.1 ± 25.2	28.7 ± 22.4	57.34 (50.34-64.35)	**<0.001**	84.2 ± 19.3	45.3 ± 24.8	38.84 (31.84-45.83)	**<0.001**	<0.001
90d-IPA	78.7 ± 26.1	26.0 ±18.2	52.65 (45.49-59.81)	**<0.001**	81.9 ± 24.0	44.7 ± 24.6	37.24 (29.08-45.41)	**<0.001**	0.006

^a^Loss-of-function allele carriers, defined as patients with at least one *CYP2C19* loss-of-function allele (ie, *2 or *3): *1/*2, *1/*3, *2/*2, *2/*3, *3/*3, *2/*17, or *3/*17.

^b^Loss-of-function non-carriers were defined as patients with no CYP2C19loss-of-function allele: *1/*1, *1/*17, or *17/*17.

Only several new-onset strokes occurred at 7 days while no myocardial infarction, death or fatal/life-threatening events happened. The incidence of new-onset stroke did not differ between ticagrelor/aspirin therapy and clopidogrel/aspirin therapy either in carriers (rates with 5.0% for ticagrelor/aspirin therapy vs 5.1% for clopidogrel/aspirin therapy; hazard ratios (HR), 0.94; 95% CI 0.71-1.25; p=0.64; Table 5) or non-carriers (rates with 1.3% for ticagrelor/aspirin therapy vs 5.8% for clopidogrel/aspirin therapy; HR, 1.01; 95% CI, 0.74-1.38; p=0.94; p=0.83 for interaction; Table 5). Only one other bleeding event occurred in carriers treated with clopidogrel/aspirin therapy. No patient developed myocardial infarction, death or life-threatening events at 7days.

**Table 5:** Major clinical events and safety of ticagrelor/aspirin as compared with clopidogrel/aspirin at 7 days stratified by metabolizer status.

	**Carriers^a^**				**Non-carriers^b^**				**p valune for interaction**
	**Ticagrelor/aspirin**	**Clopidogrel/aspirin**	**Hazard ratio (95% CI)**	**p**	**Ticagrelor/aspirin**	**Clopidogrel/aspirin**	**Hazard ratio (95% CI)**	**p**	
New-onset stroke^c^	5/100(5.0%)	5/99 (5.1%)	0.94(0.71-1.25)	0.64	1/80(1.3%)	5/86 (5.8%)	1.01(0.74-1.38)	0.94	0.83
Myocardial infarction	0/100	0/99	-	-	0/80	0/86	-	-	1.0
Death	0/100	0/99	-	-	0/80	0/86	-	-	1.0
fatal/life-threatening events	0/100	0/99	-	-	0/80	0/86	-	-	1.0
Other events	0/100	1/99 (1.0%)	-	0.31	0/80	0/86	-	-	0.96

^a^Loss-of-function allele carriers, defined as patients with at least one CYP2C19 loss-of-function allele (ie, *2 or *3): *1/*2, *1/*3, *2/*2, *2/*3, *3/*3, *2/*17, or *3/*17.

^b^Loss-of-function non-carriers were defined as patients with no CYP2C19 loss-of-function allele: *1/*1, *1/*17, or *17/*17.

^c^New-onset stroke, including ischemic and hemorrhagic stroke occurs on 7 days.

## DISCUSSION

In the present study, ticagrelor/aspirin therapy tended to have stronger platelet inhibition and more rapid onset in platelet inhibition compared with clopidogrel/aspirin therapy irrespective of *CYP2C19* metabolizer status in patients with ischemic stroke and TIA. In addition, ticagrelor/aspirin therapy in our study showed a near elimination of HOPR at 24 hours in both *CYP2C19* LOF carriers and non-carriers.

Clopidogrel as the main prescription for minor acute stroke has some limitations, such as irreversible platelet inhibition, relatively slow onset of action, and variable effect on platelet function and prognosis of stroke influenced by genetic factors, comorbidities and adjunctive pharmacotherapy. [10–12] Moreover, previous studies showed that even high-dose clopidogrel administration was not able to overcome the variability of antiplatelet effects caused by *CYP2C19* LOF alleles. [13, 14] The randomized double-blind assessment of the ONSET and OFFSET of the antiplatelet effects of ticagrelor versus clopidogrel in patients with stable coronary artery disease study firstly characterized the onset and offset of the antiplatelet effect of ticagrelor compared with clopidogrel. [15] The study showed that ticagrelor achieved a significant antiplatelet effect within 30 minutes and a greater antiplatelet effect during maintenance therapy compared with clopidogrel. Gurbel et al. investigated the antiplatelet effect of ticagrelor in clopidogrel non-responders, who received 300mg clopidogrel per day for 2 to 4 weeks before study and the absolute change in platelet aggregation was ≤10%, and found that ticagrelor had stronger platelet inhibition and was not influenced by clopidogrel response status. [16] In addition, the study indicated an additional platelet inhibition (≈20% increase in IPA) during switching from clopidogrel to ticagrelor in clopidogrel responders. Our study was consistent with previous study that ticagrelor/aspirin therapy achieved greater inhibition of platelet function and more rapid onset compared with clopidogrel/aspirin therapy in the present study.

Previous study identified patients with IPA <40% as resistant. [17] In the present study, IPAs of both carriers and non-carriers treated with ticagrelor/aspirin were much higher than 40% 2 hours after administration. While IPAs of carriers with clopidogrel/aspirin therapy were consistently lower than 40%, and non-carriers with clopidogrel/aspirin therapy had IPA increasing to nearly 40% after 24 hours, and reaching more than 40% in the 7 days after administration. Luo and his colleagues reported that the combination of an elevated PRU and a decreased IPA was associated with significantly higher incidence of major adverse cardiac events than one or neither. [18] However, the present study was not powerful enough to show whether proportion of HOPR and IPA were associated with clinical outcomes of acute minor stroke and TIA patients.

Previous study showed that *CYP2C19* LOF alleles were associated with increased risk of cardiac-cerebral vascular events and poorer clinical outcomes, [3, 19, 20] especially in Asians. [21] Kazi et al. suggested that genotype-guided personalized therapy may improve the cost-effectiveness of the newer antiplatelet agents, additionally, ticagrelor was considered to be the most cost-effective for carriers and non-carriers of *CYP2C19* LOF alleles in acute coronary disease. [22] Although the SOCRATES study did not achieve a significant result, it indicated that combination of ticagrelor and aspirin might be effective in Asian patients. [23, 24] As reported, there was a high risk of another stroke after minor stroke and TIA in the first two weeks, with particularly high events rates in the first two days. [1, 25] In our study, the ticagrelor/aspirin therapy could quickly take effect on IPA within 2 hours and almost diminished the HOPR at 24 hours both in carriers and non-carriers of *CYP2C19* LOF alleles, while clopidogrel/aspirin therapy seemed to reach maximal effect until 7 days. Our results indicated that ticagrelor/aspirin therapy might be a more appropriate strategy for acute minor stroke and TIA patients carrying *CYP2C19* LOF allele. Considering the high cost of platelet function test and expensive charge of ticagrelor, perhaps, the genotype-guided personalized combination therapy of ticagrelor and aspirin may be more cost effective. Further study on the efficacy of more intensive platelet inhibition on prognosis of acute minor stroke and TIA is needed.

Our study has several limitations. First, the number of included patients is small. The calculated number of included patients of the PRINCE study was 952 patients to achieve the primary outcome. The interim analysis based on 476 patients (50% of the projected necessary sample size) with intact data conducted by data safety monitoring board, achieved a prespecified threshold for efficacy, so the study was terminated in advance. Our study is a sub-analysis and we reviewed the previous articles on antiplatelet effects of ticagrelor and clopidogrel, the number of included patients was always between 100-200. Thus, the sample size of our study could be effective. Second, *CYP2C19* gene could also be found in other disease, which will affect platelet aggregation through other factors not only clopidogrel. It may affect the conclusion of this manuscript. Third, the present study showed that the inhibition of platelet function was maximized at 24 hours of ticagrelor/aspirin therapy (Figure 3), while previous studies showed the maximal antiplatelet effect occurred at 1-2 hours, [16, 26] which may due to the inconsecutive testing.

In conclusion, ticagrelor/aspirin therapy was associated with greater platelet inhibition and more rapid onset in platelet inhibition compared with clopidogrel/aspirin therapy both in carriers and non-carriers of *CYP2C19* LOF alleles with acute minor stroke or TIA.

## MATERIALS AND METHODS

### Study participants and protocol

We derived data from the PRINCE trial. Details on design and major results of the PRINCE trial have been published elsewhere. [27] Briefly, it was a prospective, multicenter, randomized, open-label, active-controlled and blinded-endpoint trial conducted in China compared ticagrelor (loading dose of 180 mg followed by 90 mg twice daily till day 90) combined with aspirin (loading dose of 100-300mg followed by 100 mg once daily till day 21) and clopidogrel (loading dose of 300mg followed by 75 mg daily till day 90) combined with aspirin (loading dose of 100-300mg followed by 100 mg once daily till day 21) among 675 patients with acute minor stroke defined as National Institutes of Health Stroke Scale score of ≤3 or those with a moderate to high risk TIA defined as ABCD^2^ stroke risk score of ≥4 or ≥50% stenosis of cervical or intracranial vessel that was responsible for the presentation within 24 hours of symptom onset. Participants were from 26 hospitals in China between August 2015 and March 2017 to estimate whether ticagrelor/aspirin therapy was safe and superior to clopidogrel/aspirin therapy in inhibiting the 90-day platelet reactivity. The protocol and data collection were approved by ethics committee of Beijing Tiantan Hospital and all participated study centers. All patients or their representatives provided written consent before enrollment.

Considering that CYP2C19 gene can affect many other P450 metabolized drugs, if the patient is taking a drug affected by the CYP2C19 genotype in prior to enrollment, we will recommend stopping the drug or switching to an alternative drug. Detailed information of drug combination was collected. And such patients will be labeled and treated accordingly. All medicine including Chinese herbal medicine that may affect function of platelet is prohibited during the trial. Other medicine such as lipid-lowering medicine, antidiabetic, antihypertensive drugs, can be normally used.

The early test sub-study was pre-specified. Patients included in the sub-study additionally received VerifyNow P2Y12 assay testing 2 hours and 24 hours after taking the first agents (Figure 1).

The primary outcomes of our study were the proportions of patients with HOPR and IPA at baseline, 2 hours, 24 hours, 7 days and 90 days, respectively. The HOPR was defined as PRU>208. Percentage of IPA was calculated using standard formulas, where PA was platelet aggregation, b was pre-dosing, and t was post-dosing: IPA (%) = 100% × (PA_b_ – PA_t_)/PA_b_.

The primary safety outcome was major clinical events including new-onset ischemic or hemorrhagic stroke, myocardial infarction, death, and major bleeding according to the Platelet Inhibition and Patient Outcomes (PLATO) study including fatal/life-threatening bleed, major bleed and others, at 7 days.

The PRU and IPA are tested by the VerifyNow P2Y12 assay, a turbidimetric-based optical detection system, according to the manufacturer’s instructions. The VerifyNow P2Y12 assay was well studied and used widely in testing P2Y12 receptor activity. The device measured platelet-induced aggregation as an increase in light transmittance and used a proprietary algorithm to report values of PRU. [28]

### Genotyping

Three single-nucleotide polymorphisms for *CYP2C19* (National Center for Biotechnology Information [NCBI] Genome build 37.1, GenBank NG_008384), including CYP2C19*2 (681G>A, rs4244285), CYP2C19*3 (636G>A, rs4986893), and CYP2C19*17 (-806C>T, rs12248560), were genotyped in all participants recruited. Details of genotyping were published. [9]

We used common consensus star allele nomenclature to categorize patients by *CYP2C19* metabolizer status based on *2, *3, and *17 genotypes. [29] Gain-of-function allele carriers were defined as who had at least one gain-of-function allele (*17) and LOF allele carriers were defined as who had at least one LOF allele (*2 or *3). [3] Patients with at least two *2 or *3 alleles (*2/*2, *2/*3, or *3/*3) were classified as "poor metabolizers", those with one *2 or *3 allele (*1/*2 or *1/*3) were classified as "intermediate metabolizers", those with at least one *17 allele (*1/*17 or *17/*17) were classified as "ultra-metabolizers", those without any *2, *3, or *17 allele (*1/*1) were classified as "extensive metabolizers", and those with one *17 and a LOF allele (*2/*17 or *3/*17) were classified as "unknown metabolizers" [30] due to the uncertain clinical consequences. [31]

### Statistical analysis

All statistical analysis was performed using the SAS 9.4 (SAS Institute Inc, Cary, NC). All reported p values were two-sided with p<0.05 considered significant. The baseline characteristics were compared between patients included and excluded, and between two treatment groups in carriers and non-carriers of *CYP2C19* LOF alleles. Categorical variables were presented in proportions, and continuous variables were presented in means ±SD or medians with interquartile ranges. Nonparametric Kruskal-Wallis test was used to compare group differences for nominal variables, and χ2 tests or Fisher’s exact test for dichotomous variables. The proportions of HOPR and IPAs at 2 hours, 24 hours, 7 days and 90 days were respectively compared between the two therapy groups using genmod models adjusted by the high platelet reactivity status at baseline, RR with 95% CI was presented for HOPR, and β with 95% CI was presented for IPA. Differences in the rates of new-onset stroke, myocardial infarction, death, and bleeding events during 7-day follow-up were assessed using Cox proportional hazards regression and HR with 95% CI. Whether the treatment effect differed in certain genotype categories was assessed by testing the treatment-by-genotype interaction effect using genmod models for the primary outcome and Cox models for the primary safety outcome.
